# Supplementary figures and images for: A novel MARV glycoprotein-specific antibody with potentials of broad-spectrum neutralization to filovirus
Source: eLife. 2024 Mar 25;12:RP91181. doi: 10.7554/eLife.91181 (PMC10963030; doi:10.7554/eLife.91181)

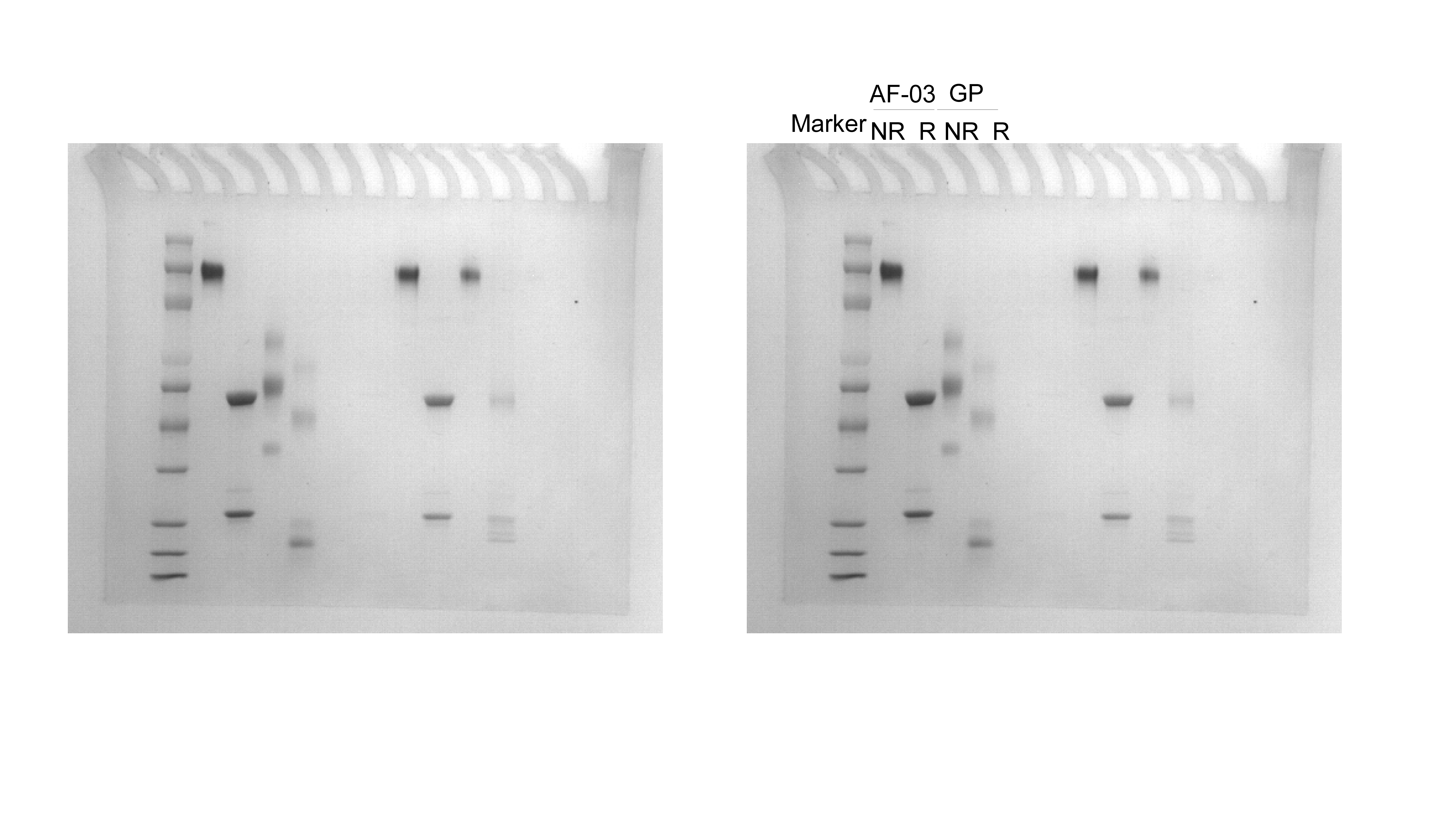

Supplement: Figure 1—source data 1. [file elife-91181-fig1-data1.zip › Fig.1 source data/Figure 1A.tif]

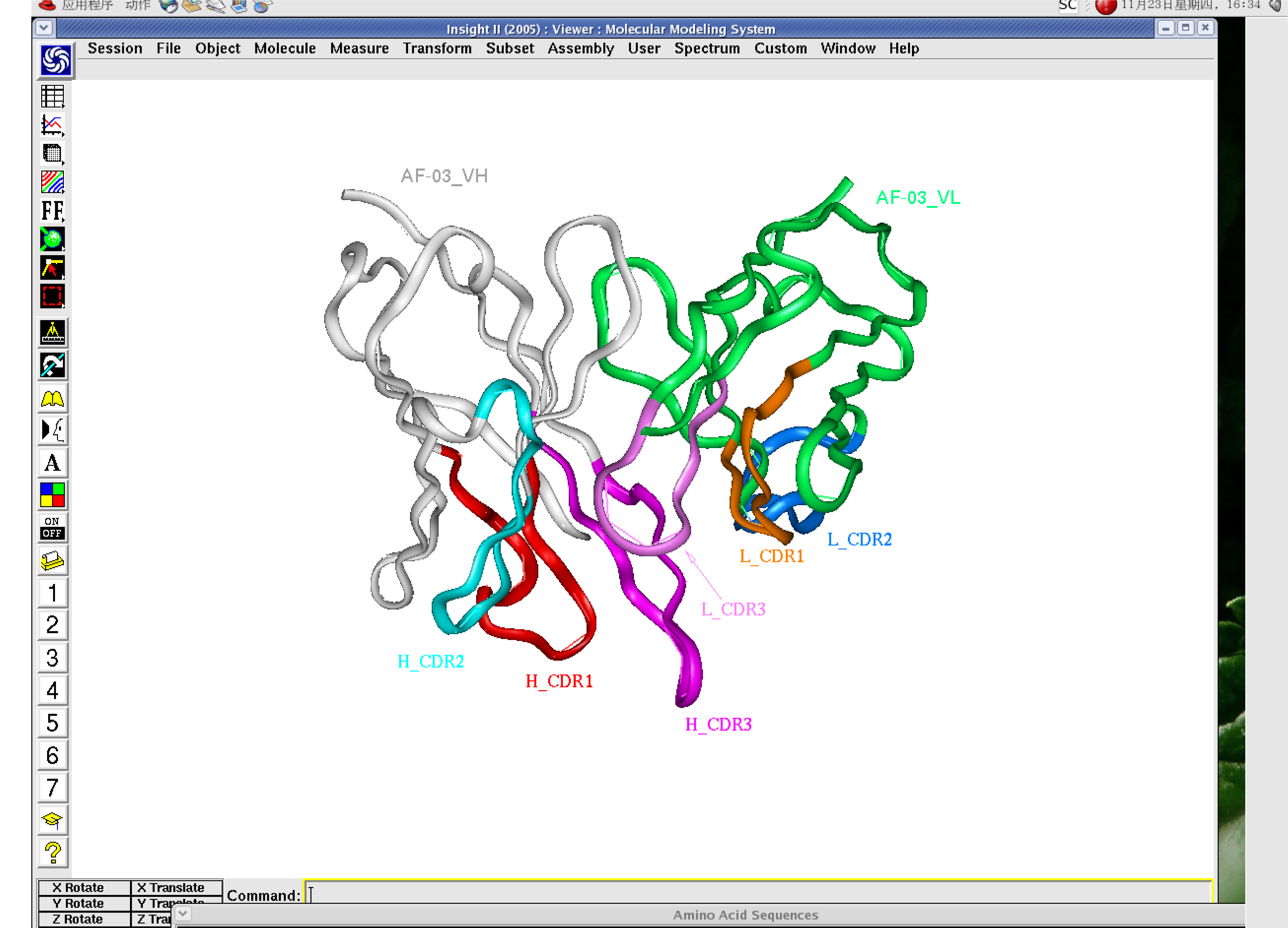

Supplement: Figure 1—source data 1. [file elife-91181-fig1-data1.zip › Fig.1 source data/Figure 1D.tif]

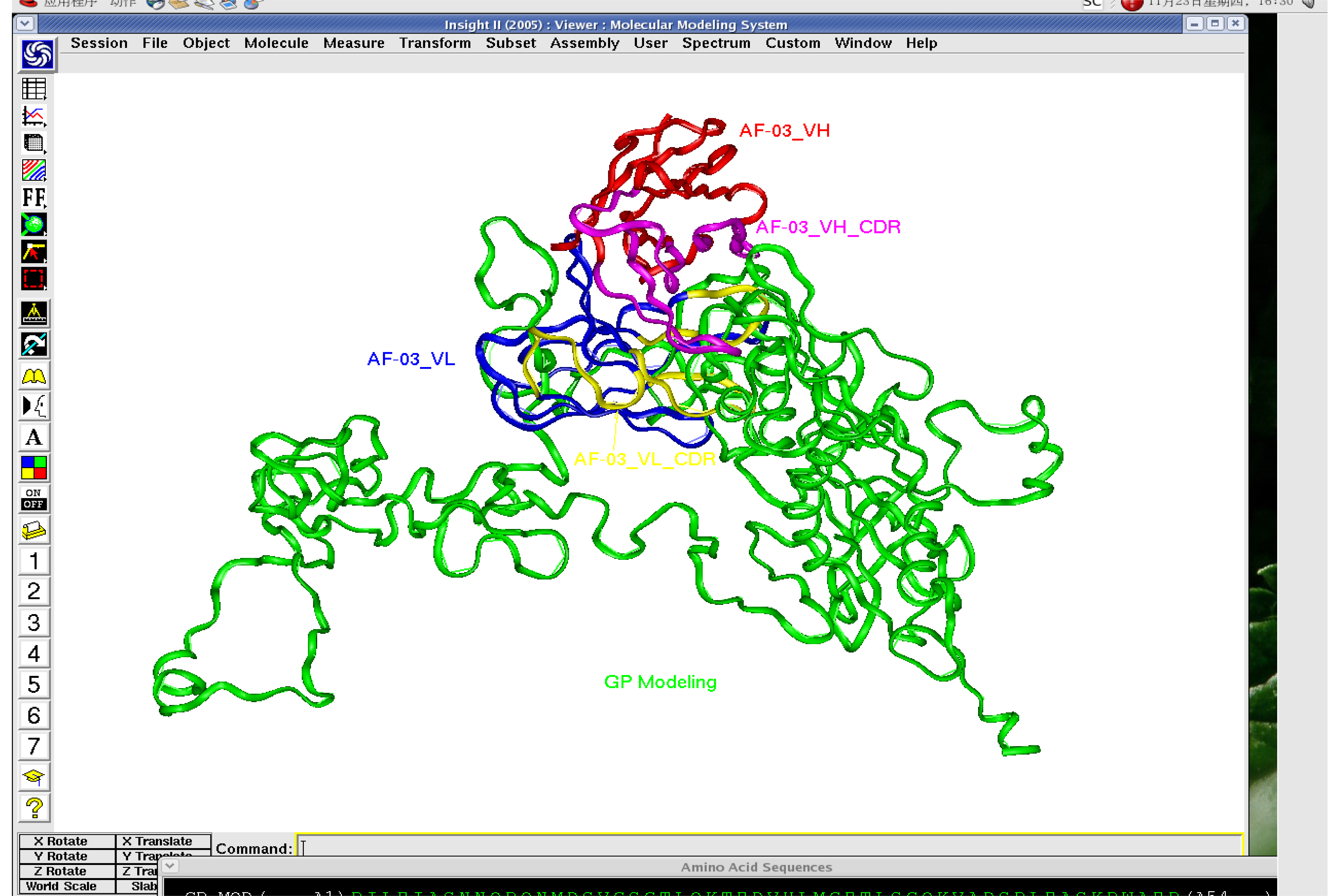

Supplement: Figure 1—source data 1. [file elife-91181-fig1-data1.zip › Fig.1 source data/Figure 1E.tif]

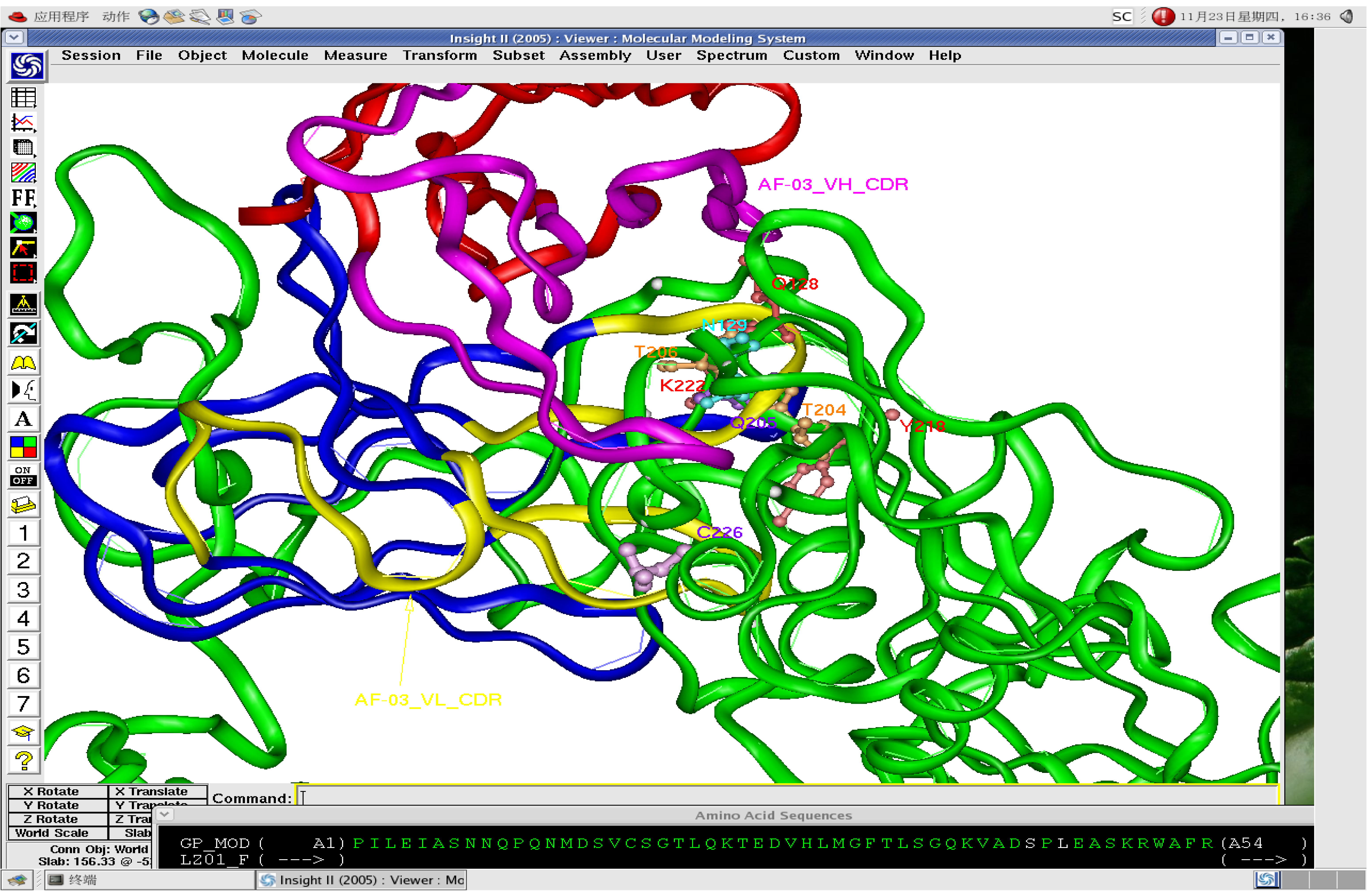

Supplement: Figure 1—source data 1. [file elife-91181-fig1-data1.zip › Fig.1 source data/Figure 1F.tif]

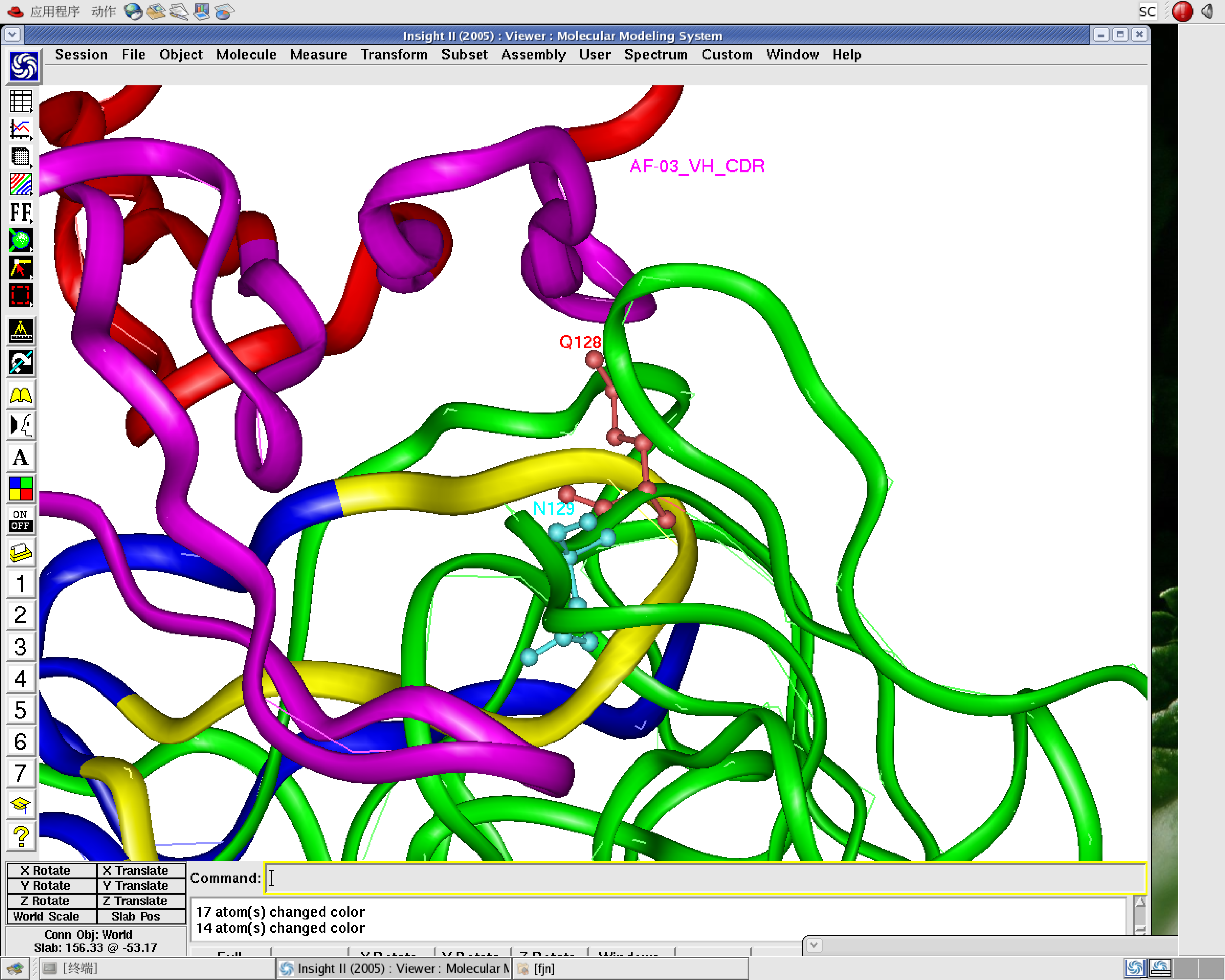

Supplement: Figure 1—source data 1. [file elife-91181-fig1-data1.zip › Fig.1 source data/Figure 1F-2.tif]

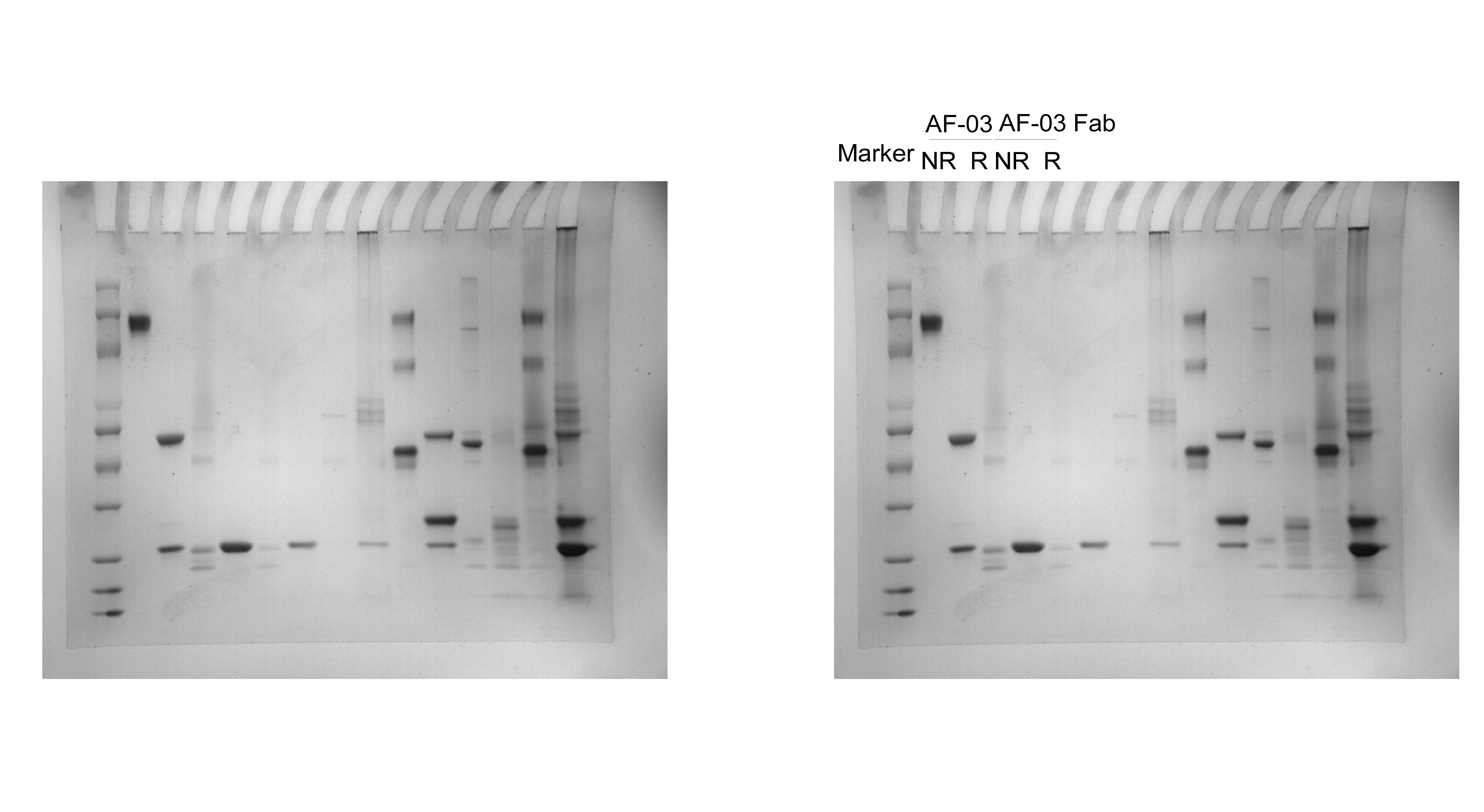

Supplement: Figure 1—figure supplement 1—source data 1. [file elife-91181-fig1-figsupp1-data1.zip › Figure 1-figure supplement 1 source data/Figure 1-figure supplement 1A.tif]

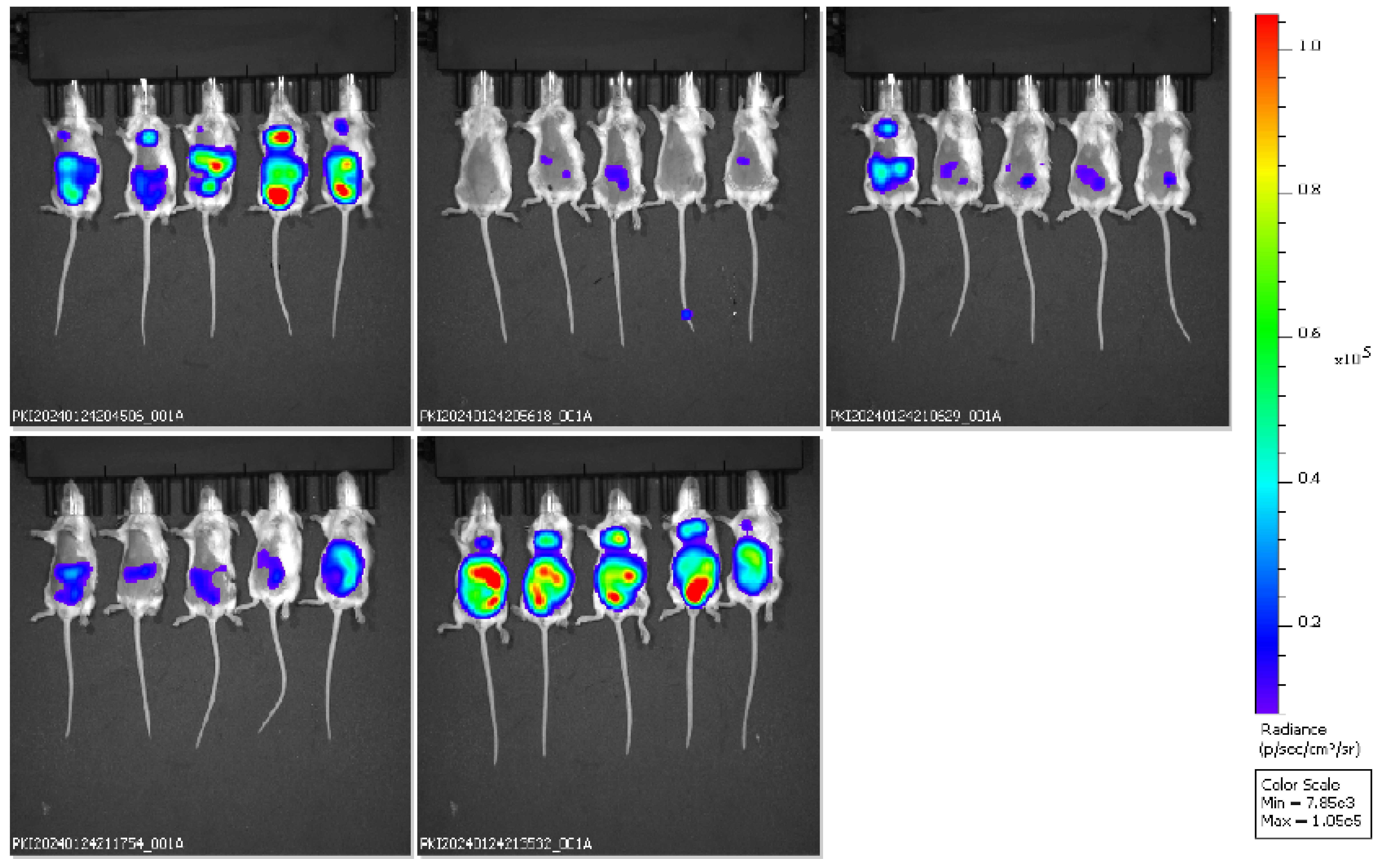

Supplement: Figure 3—source data 1. [file elife-91181-fig3-data1.zip › Fig.3 source data/Figure 3C.tif]

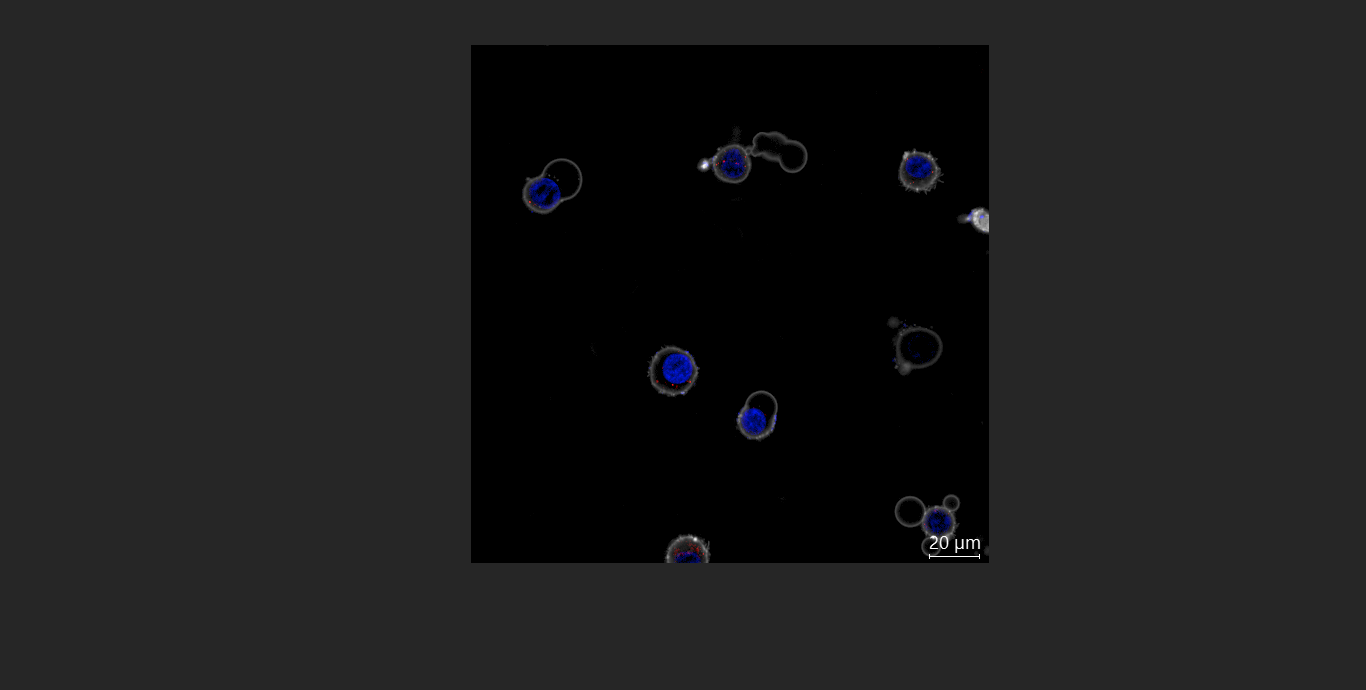

Supplement: Figure 5—source data 1. [file elife-91181-fig5-data1.zip › Fig.5 source data/Fig.5C/AF-03.tif]

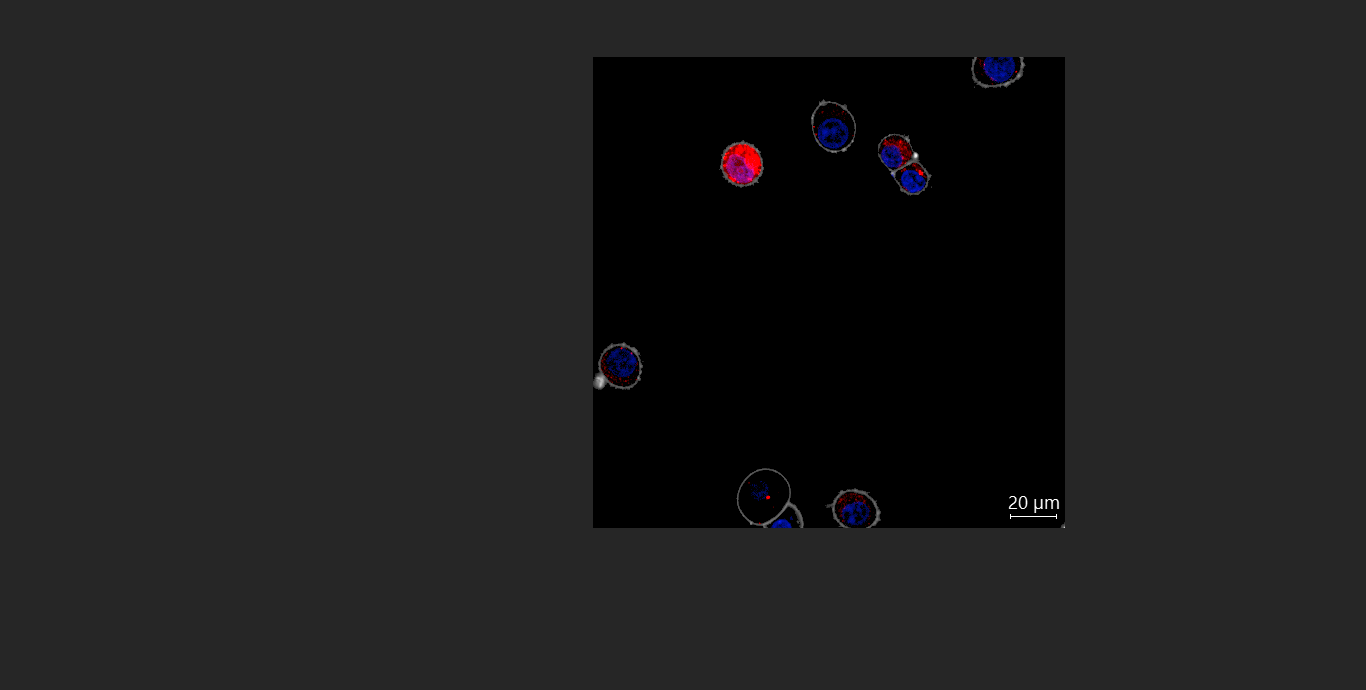

Supplement: Figure 5—source data 1. [file elife-91181-fig5-data1.zip › Fig.5 source data/Fig.5C/AF03-NL.tif]

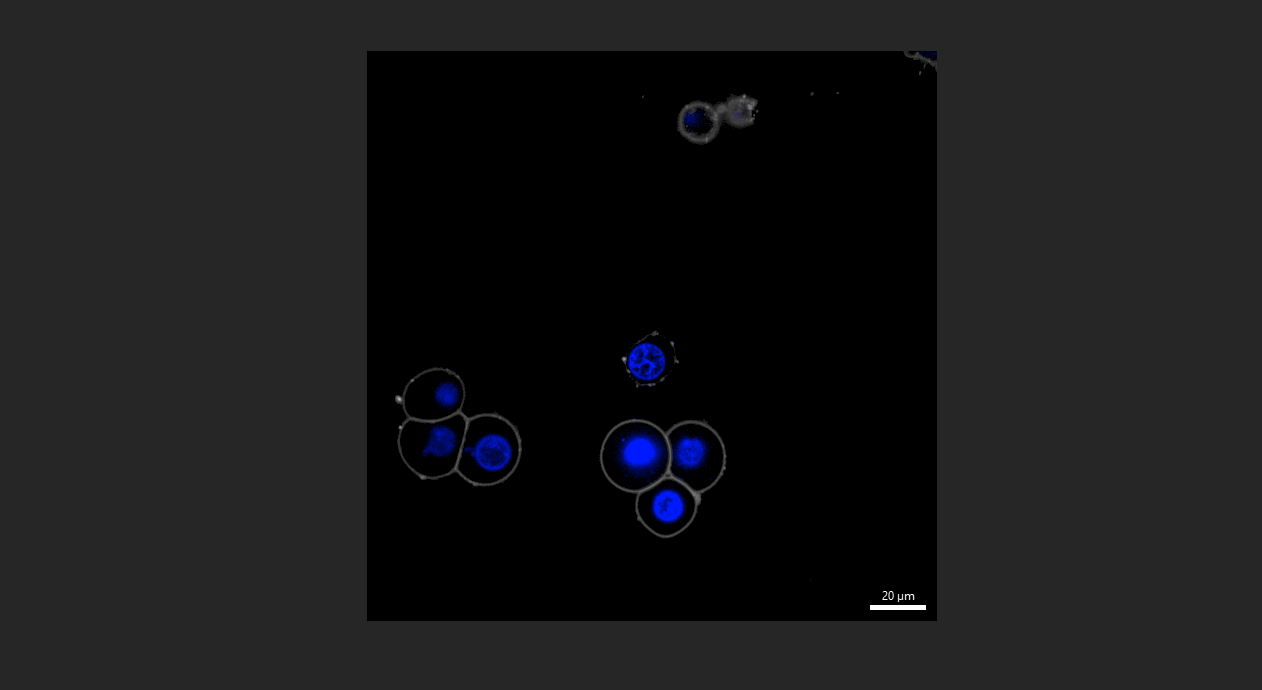

Supplement: Figure 5—source data 1. [file elife-91181-fig5-data1.zip › Fig.5 source data/Fig.5C/Mock.tif]

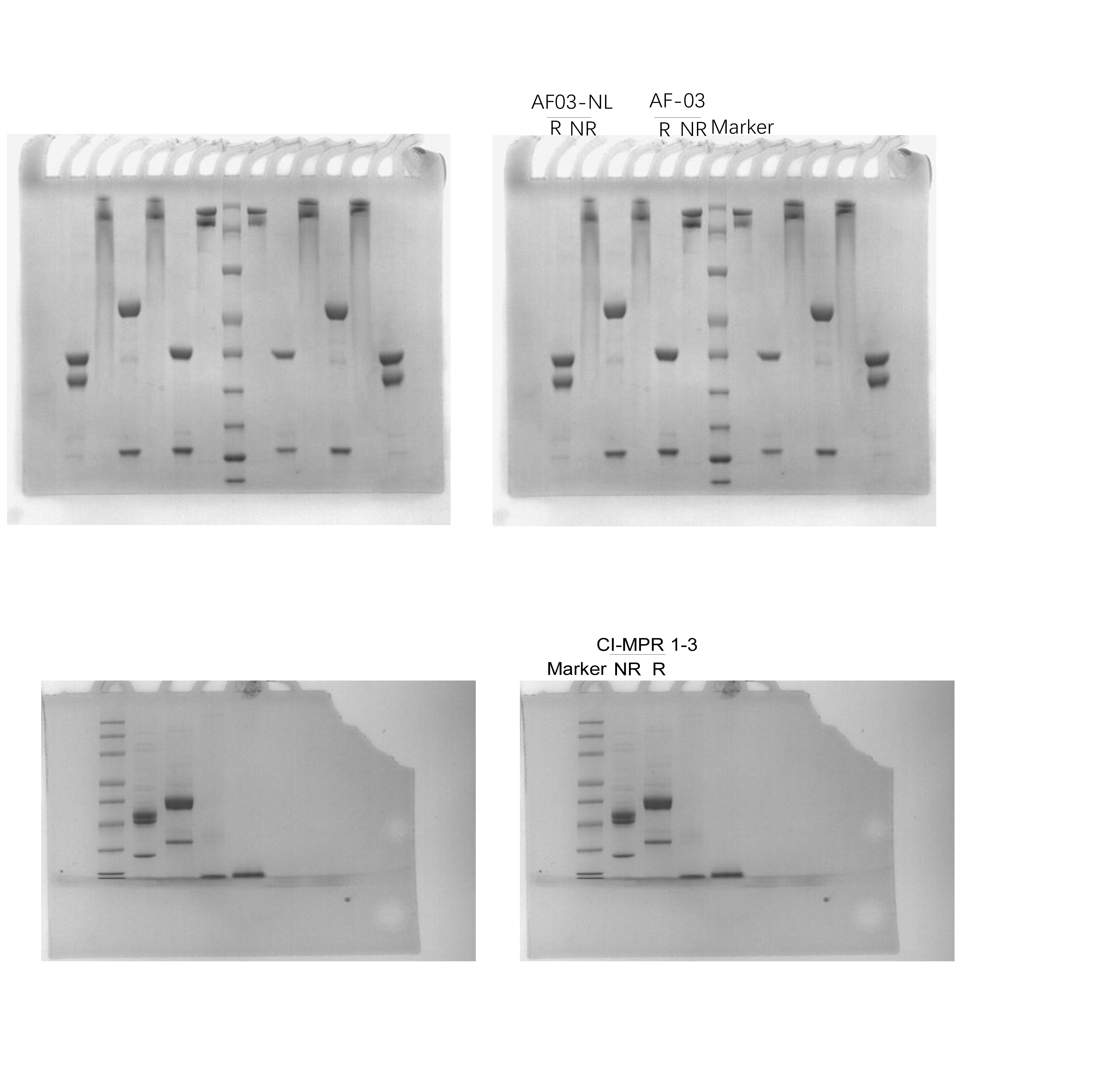

Supplement: Figure 5—figure supplement 1—source data 1. [file elife-91181-fig5-figsupp1-data1.zip › Figure 5-figure supplement 1 source data/Figure 5-figure supplement 1A.tif]

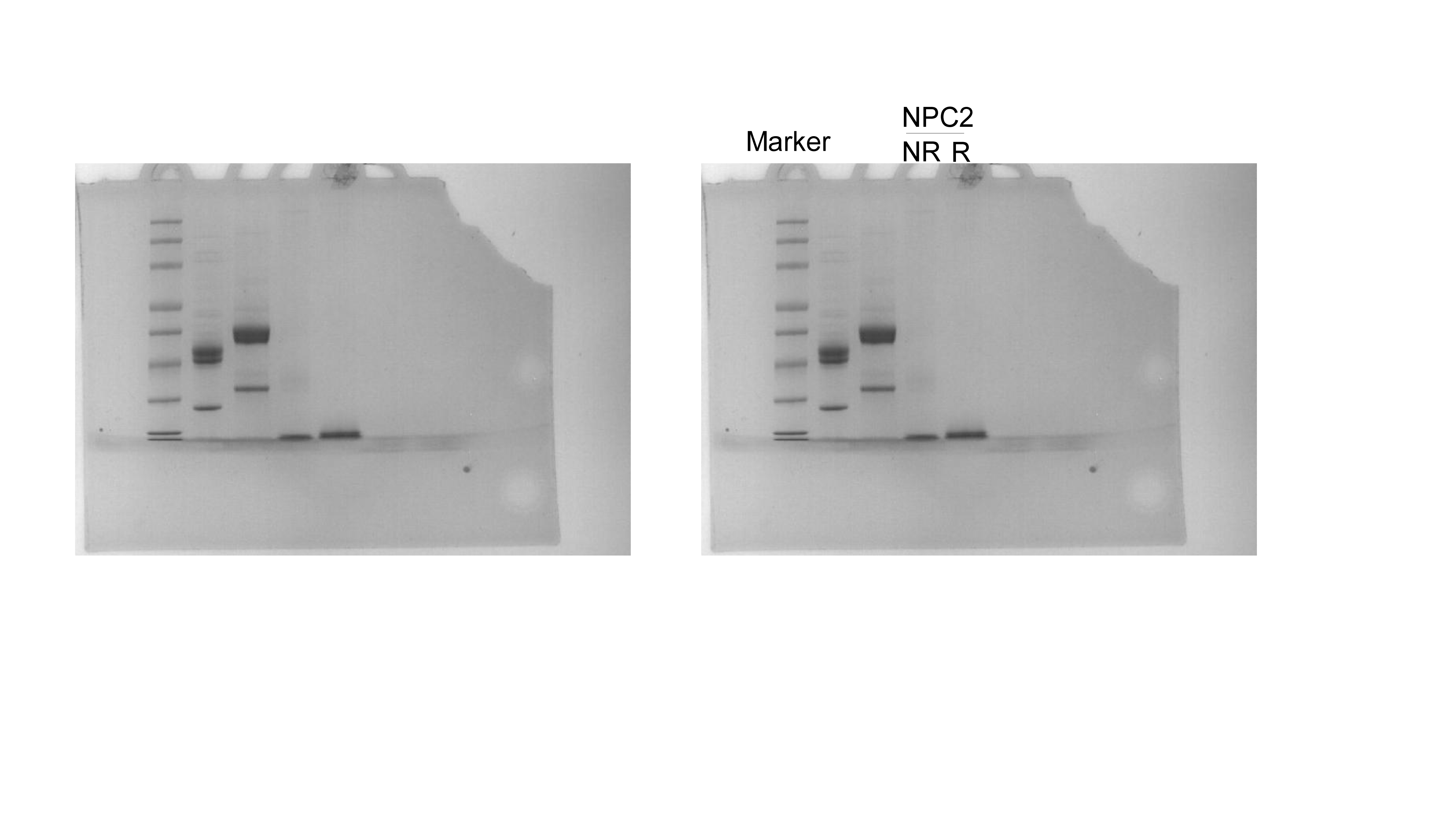

Supplement: Figure 7—source data 1. [file elife-91181-fig7-data1.zip › Fig.7 source data/Figure 7A.tif]
