# Supplementary material for: A novel MARV glycoprotein-specific antibody with potentials of broad-spectrum neutralization to filovirus
Source: eLife. 2024 Mar 25;12:RP91181. doi: 10.7554/eLife.91181 (PMC10963030; doi:10.7554/eLife.91181)
Supplement: Supplementary file 1. [file elife-91181-supp1.docx]

**Supplementary Table**

Tab.s1 Proportion of secondary structure of parental and mutant MARV GP

|  | MARV GP | Q^128^S-N^129^S | C^226^Y |
| --- | --- | --- | --- |
| Helix | 18.15% | 19.46% | 19.28% |
| Antiparallel | 13.66% | 12.95% | 13.03% |
| Parallel | 12.10% | 11.78% | 11.85% |
| Beta-Turn | 18.61% | 18.44% | 18.39% |
| Rndm. Coil | 37.49% | 37.37% | 37.45% |
| Total Sum | 100.00% | 100.00% | 100.00% |

The parental MARV GP and mutants were purified and replaced by PBS ultra-filtration. The secondary structure of the protein was analyzed by Circular Dichroism.
